# Metabolomic analysis reveals key metabolites alleviating green spots under exogenous sucrose spraying in air-curing cigar tobacco leaves

**DOI:** 10.1038/s41598-023-27968-8

**Published:** 2023-01-24

**Authors:** Nanfen Li, Jun Yu, Jinpeng Yang, Sheliang Wang, Lianying Yu, Fangsen Xu, Chunlei Yang

**Affiliations:** 1 Microelement Research Center, College of Resource and Environment, Huazhong Agricultural University, Wuhan, China; 2 Tobacco Research Institute of Hubei Province, Wuhan, China

**Keywords:** Biological techniques, Biotechnology, Physiology, Plant sciences

## Abstract

Cigar variety CX-010 tobacco leaves produce localized green spots during the air-curing period, and spraying exogenous sucrose effectively alleviates the occurrence of the green spots. To investigate the alleviation effect of exogenous sucrose spraying, the total water content and the number and size of green spots on tobacco leaves were investigated during the air-curing period under four treatments; CK (pure water), T1 (0.1 M sucrose), T2 (0.2 M sucrose) and T3 (0.4 M sucrose). The results showed that the total water content of tobacco leaves showed a trend of T3 < CK < T2 < T1 in the early air-curing stage, and the number and size of green spots showed a trend of T3 < T2 < T1 < CK. All sucrose treatments alleviated the green spot phenomenon, and T3 had the fewest green spots. Thus, the tobacco leaves of the T3 and CK treatments at two air-curing stages were used to perform metabolomics analysis with nontargeted liquid chromatography‒mass spectrometry to determine the physiological mechanism. A total of 259 and 178 differentially abundant metabolites (DAMs) between T3- and CK-treated tobacco leaves were identified in the early air-curing and the end of air-curing stages, respectively. These DAMs mainly included lipid and lipid-like molecules, carbohydrates, and organic acids and their derivatives. Based on the Kyoto Encyclopedia of Genes and Genomes (KEGG) pathway analysis, the T3 treatment significantly altered carbohydrate metabolism (pentose phosphate pathway, sucrose and starch metabolism and galactose metabolism) and amino acid metabolism (tyrosine metabolism and tryptophan metabolism) in air-curing tobacco leaves. Sucrose treatment alleviated green spots by altering DAMs that affected chlorophyll degradation, such as tyrosine and citric acid, to promote the normal degradation of chlorophyll.

## Introduction

In the rapidly growing cigar tobacco market, the shortage of quality raw materials for cigar tobacco in China has become a limiting factor for cigar products and the industry^1^. Cigar tobacco (*Nicotiana tabacum* L.) is essentially dark air-cured tobacco, and its quality is influenced by the variety, cultivation techniques, harvesting at maturity, air-curing and fermentation conditions. Air-curing refers to the processing of the harvested tobacco leaves in natural or artificial air-curing rooms with controlled temperature, humidity, and ventilation (and sunlight). This makes sure that the air-cured tobacco leaves have a consistent color and a harmonious ratio of sugar, nicotine, and other components to enhance the industrial viability of tobacco leaves^2,3^. The air-curing of cigar tobacco is a slow process of water loss accompanied by a slow change in color. The color appearance is a key factor of wrapper leaves during the production process because it can be visually evaluated by consumers. Cigar tobacco leaves harvested from Enshi, Hubei Province, China, had a normal appearance except that the wrapper variety CX-010 showed partial tissue necrosis (named “green spots”) at the early air-curing stage. Our previous study^4^ showed that chlorophyll degradation was impaired in green spots, and amino acids and polyphenols were not converted properly. It is suspected that the occurrence of green spots in the early air-curing stage of tobacco leaves may be related to some kind of stress.

The addition of exogenous sugars can trigger different sugar signalling pathways or directly regulate starch, lipid metabolism, stress-related osmoregulatory substances, and other metabolic pathways and subsequently improve plant resistance to biotic as well as abiotic stresses^5–7^. Many studies have reported a strong correlation between sucrose concentration and stress tolerance^8–10^, indicating that plants have developed an effective sensing and transduction system induced by low or high sugar concentrations. Sucrose is involved in both ROS production pathways, such as the regulation of mitochondrial respiration or photosynthesis, and NADPH production pathways, such as the oxidized pentose phosphate pathway, in plants are exposed to stress^11,12^. De Pascali et al. found that susceptible varieties of olive trees had a remarkable decline in sucrose content, whereas the resistant varieties of olive trees had a significant increase in sucrose content under pathogen and drought stresses^13^.

Sucrose has little effect on the processing of tobacco leaves in industrial production^14,15^, and numerous studies have shown that sucrose regulates the formation of anthocyanins^5,16,17^, which presumably play a role in the conversion of leaf color. In addition, Xie et al. showed that spraying six plant polysaccharides significantly altered the water content of cigarettes^18^. Therefore, the use of exogenous sucrose might be an effective way to eliminate the occurrence of green spots in tobacco leaves during the early air-curing period. Metabolomics can be used to identify the differential metabolites and the metabolic pathways that change in plants exposed to various environmental stimuli. Wu et al. performed metabolomics analysis in blueberry leaves under different shade treatments and found that metabolites were significantly enriched in the biosynthetic pathways of flavonoids and flavonols, providing strong evidence for the optimization of summer cultivation conditions and intensive management of blueberries^19^. Yang et al. investigated transcriptional and metabolic differences of oilseed tea tree (*Ca. oleifera*) in response to anthracnose and found that secondary metabolites such as epicatechin, proanthocyanidin B2, and arachidonic acid had important effects on resistance to anthracnose and that flavonoid biosynthesis may play an important role in the fight against anthracnose^20^. Based on metabolomics analysis, we previously reported that the differentially abundant metabolites (DAMs) in green spots compared to normal tissues were lipids, amino acids, peptides and their analogues, alkaloids, and organic acids^4^. Here, we found that spraying exogenous sucrose had a positive effect on limiting the occurrence of green spots in cigar tobacco CX-010. Furthermore, we performed a metabolomics analysis to estimate the differences in cigar leaves with/without sucrose treatments. Some DAMs were identified, and the key metabolic pathways were distinguished, which may provide a novel understanding of how sucrose limits the occurrence of green spots in cigar leaves.

## Results

### Effect of exogenous sucrose treatment on green spots on tobacco leaves

**Figure 1:**
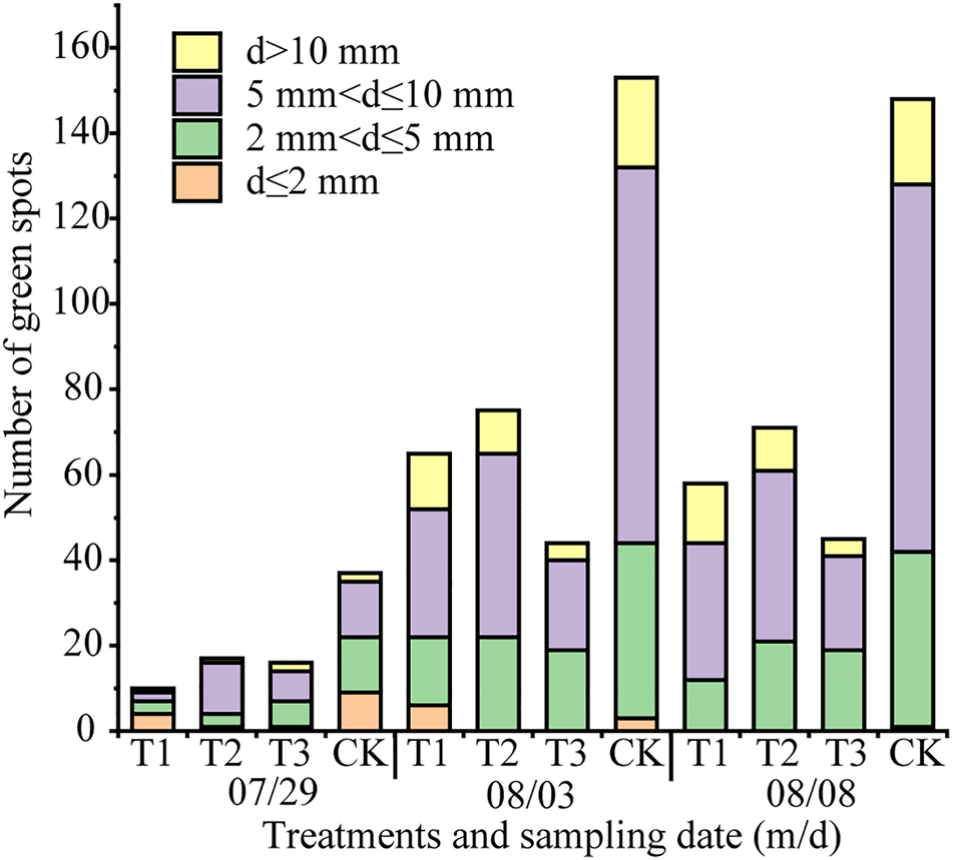
Changes in the number and size of green spots on tobacco leaves in different treatments during the air-curing period. T1 (0.1 M sucrose), T2 (0.2 M sucrose), T3 (0.4 M sucrose) and CK (pure water). Values are the number of green spots in a total of 9 tobacco leaves.

**Figure 2:**
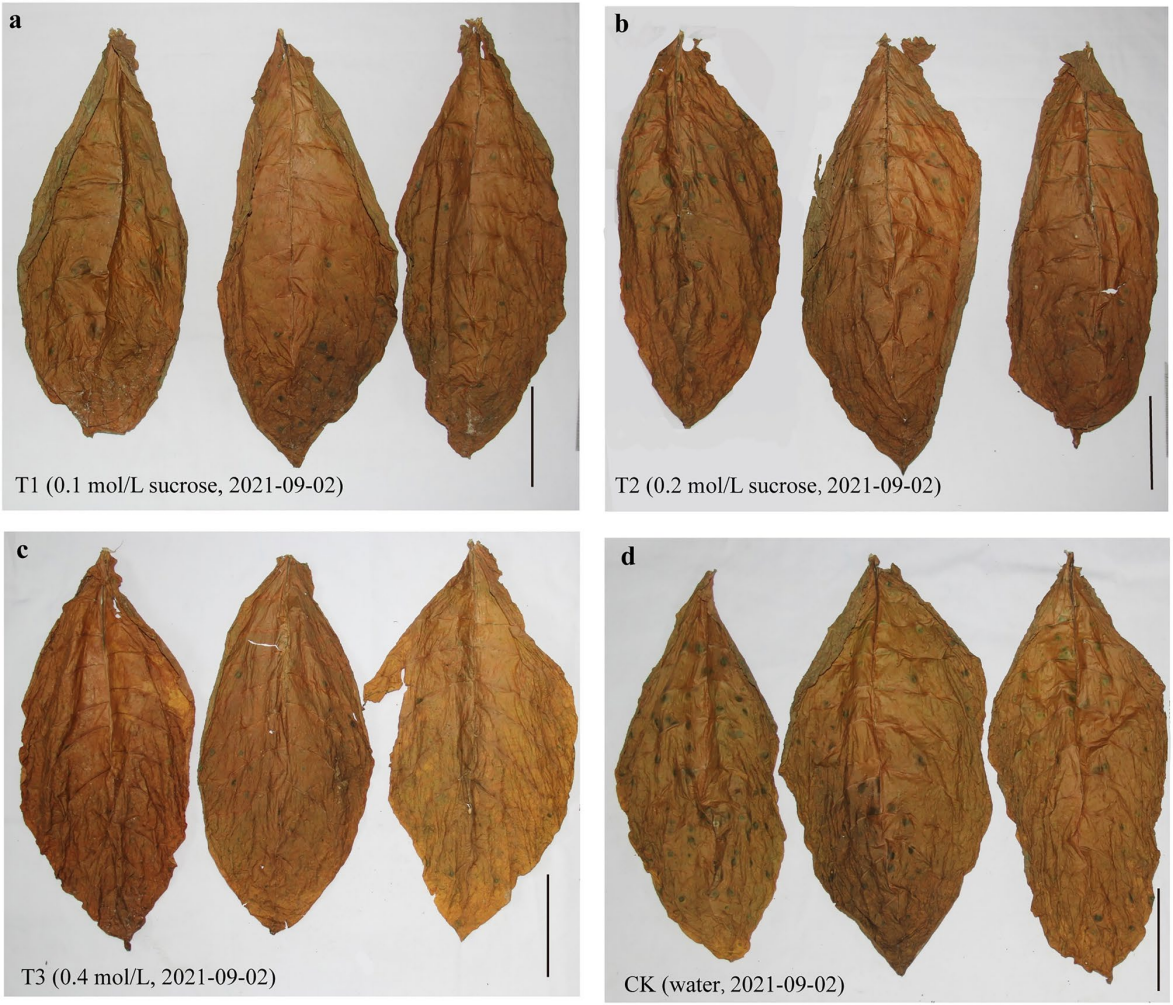
The appearance of tobacco leaves under different treatments at the end of air-curing. (**a**–**d**) for the T1, T2, T3 and CK treatments; bar = 15 cm.

The tobacco leaves harvested from the trial field were normal with uniform color and no disease spots or other undesirable conditions; however, during the early air-curing stage, the leaves formed green spots, which seriously affected cigar quality. The microstructure of green spotted tobacco leaves and normal tobacco leaves (Supplementary Fig. 1) showed that green spotted tobacco leaves were significantly thinner than normal tobacco leaves, and there were fewer grana thylakoid lamellae in the cells. To eliminate the occurrence of green spots, a spraying application of sucrose was conducted using 0.1 M (T1), 0.2 M (T2) and 0.4 M (T3) concentrations. A treatment of pure water was used as a control (CK). The number and size of green spots increased from July 29th to August 3rd on the air-curing days (Fig. 1). However, there were fewer and smaller spots under the exogenous sucrose treatments than under the CK. The number of green spots with a diameter of 5–10 mm was the greatest, accounting for almost 50%, followed by green spots with a diameter of 2–5 mm. There were almost no green spots with a diameter of less than 2 mm at the end of air-curing. The cigar leaves treated with sucrose had fewer green spots at the end of air-curing (Fig. 2), suggesting that the sucrose treatment was effective in reducing the green spots.

### Effect of exogenous sucrose treatment on the total water content of tobacco leaves during air-curing

**Figure 3:**
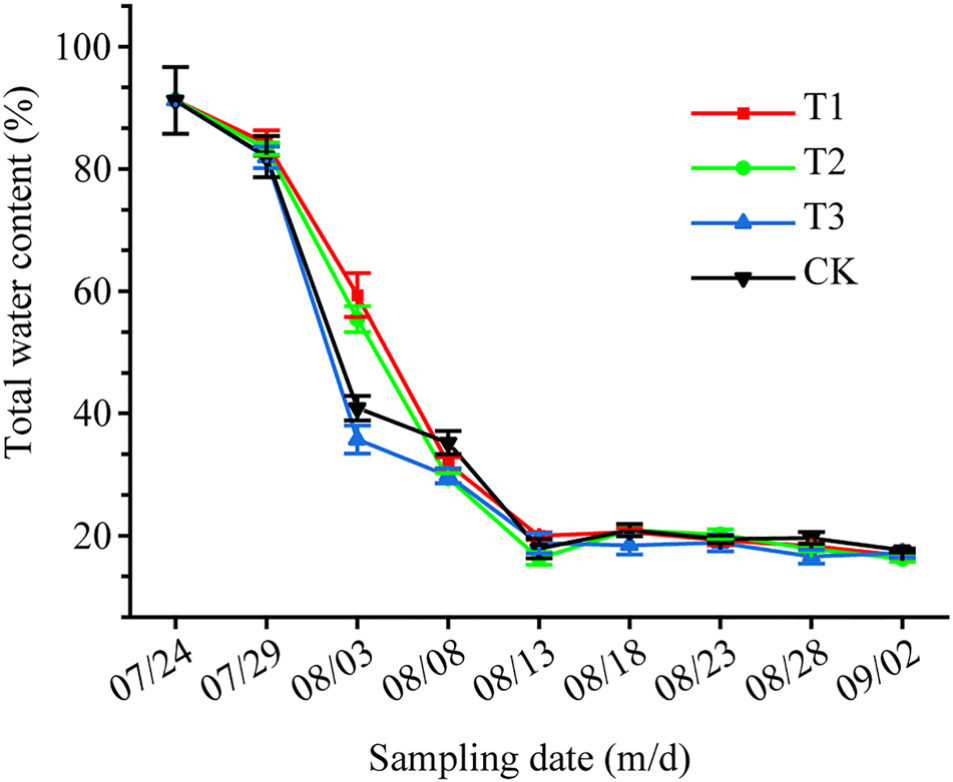
Changes in the total water content of tobacco leave in different treatments during the air-curing period. T1 (0.1 M sucrose), T2 (0.2 M sucrose), T3 (0.4 M sucrose) and CK (pure water). Values are the mean of three biological replicates.

The total water content of cigar tobacco leaves in different treatments was measured during the air-curing period. The results showed that the water content of fresh tobacco leaves was high up to 91.21% on July 24th, and the water content of each treatment decreased drastically from the 5th to 10th days of air-curing (Fig. 3), which coincided with the highest amount of green spots. The leaves in the T3 treatment lost water the fastest in the early stage before August 3rd, with the highest water loss rate of 56.41%. This result suggests that sucrose concentrations had distinct effects on the total water content of cigar leaves during the air-curing period.

### Metabolomics analysis

#### Principal component analysis (PCA)

**Figure 4:**
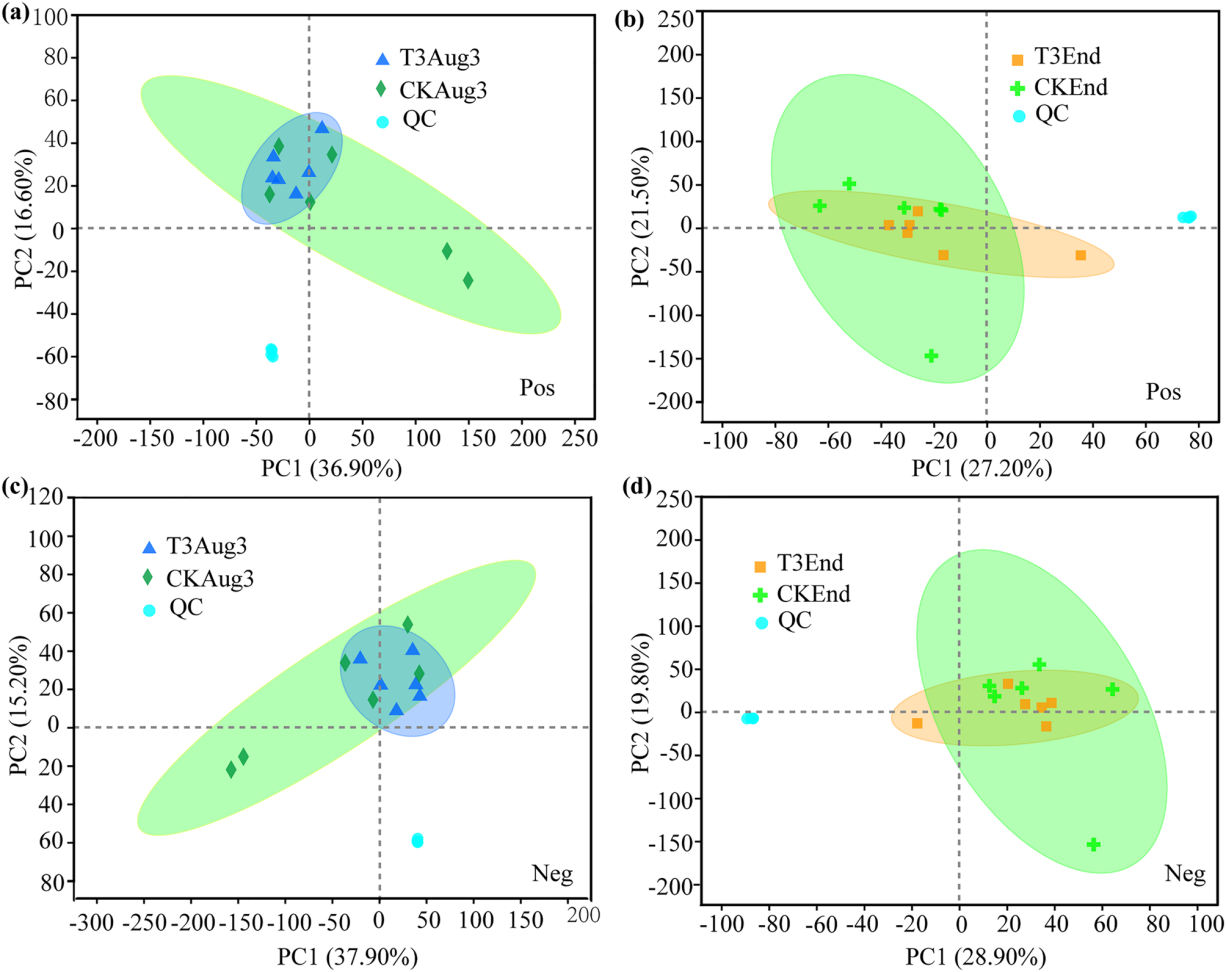
Principal component analysis of T3Aug3 vs. CKAug3 in positive (**a**) and negative (**c**) ion modes, T3End vs. CKEnd in positive (**b**) and negative (**d**) ion modes. T3Aug3 (samples from the T3 treatment on August 3rd), CKAug3 (samples from the CK treatment on August 3rd), T3End (samples from the T3 treatment on September 2nd) and CKEnd (samples from the CK treatment on September 2nd).

The above results show that the T3 treatment (0.4 M sucrose) was much more effective in managing the occurrence of green spots than the other two treatments during air-curing. In addition, according to the changes in total water content of the tobacco leaves during air-curing, the time with the greatest change in the water content was around August 3rd (Fig. 3). Therefore, the leaf samples of the T3 and CK treatments on August 3rd (the 10th day of air-curing) and September 2nd (the last day of air-curing) were selected to conduct comparative metabolite analysis to analyze the physiological mechanism of green spot development among different treatments and air-curing periods. According to the qualitative metabolite results, a total of 19,636 peaks were detected in tobacco leaves from the T3Aug3 (samples from the T3 treatment on August 3rd), CKAug3 (samples from the CK treatment on August 3rd), T3End (samples from the T3 treatment on September 2nd) and CKEnd (samples from the CK treatment on September 2nd) groups, including 9093 in positive ion mode and 10,543 in negative ion mode. Furthermore, a total of 1368 metabolites were identified, which included 592 in positive ion mode and 776 in negative ion mode. PCA can be used to observe the segregation trend between groups and within groups for the experimental model based on the original data. The results showed that, regardless of the stage (early air-curing or the end of air-curing), the distance between different groups of samples was large on the horizontal coordinate score plot, and the confidence intervals of the six biological replicate samples within the group were within the 95% confidence interval (Fig. 4), indicating that the intragroup reproducibility was good and the samples were significantly different between the groups with different treatments at different periods.

#### Analysis of differentially abundant metabolites between different treatments

**Figure 5:**
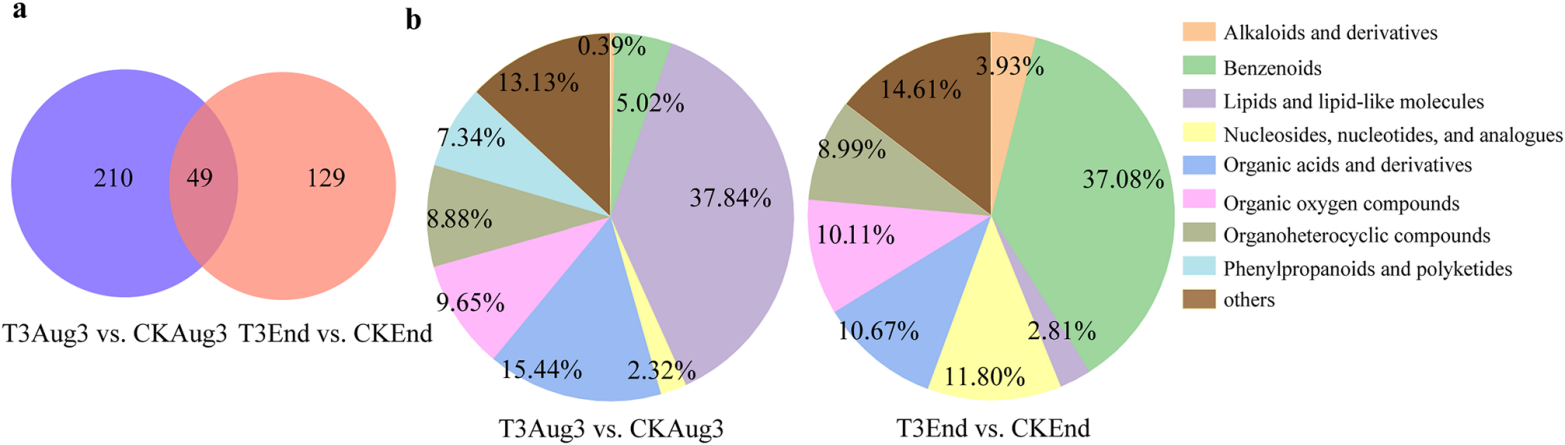
Statistical and analytical charts of differentially abundant metabolites (DAMs). (**a**) Venn diagram of DAMs in 2 comparison groups, (**b**) Pie chart of DAMs classification. T3Aug3 (samples from the T3 treatment on August 3rd), CKAug3 (samples from the CK treatment on August 3rd), T3End (samples from the T3 treatment on September 2nd) and CKEnd (samples from the CK treatment on September 2nd).

Metabolites with both a significant level of variance (*P* < 0.05, t test) and vriable importance in the projection (VIP) > 1 are considered to be taxonomically relevant and important metabolites, and those meeting this condition are called differentially abundant metabolites (DAMs). There were 49 identical DAMs in the two comparison groups (Fig. 5a). In the T3Aug3 vs. CKAug3 group, 259 DAMs (Supplementary Table 1) with names in the database were identified, where 77.22% of DAMs showed an increase in abuandance. A total of 178 DAMs were identified in the T3End vs. CKEnd group (Supplementary Table 2), where 61.24% of DAMs showed an increase. Based on the superclass of HMDB, we classified and counted the DAMs (Fig. 5b) and found that the categories and proportions of DAMs were basically the same in both comparison groups, and the most diverse DAMs were lipids and lipid-like molecules, followed by organic acids and their derivatives, and organic oxygen compounds.

#### Analysis of KEGG functions and pathways of DAMs

To understand the biological functions of the DAMs in this study, we used the KEGG database to classify DAMs in the two pairs of comparison groups according to the pathways in which they were involved or the functions they performed. The results showed that the effects of T3 treatment on tobacco leaves were mainly on carbohydrate metabolism, amino acid metabolism and lipid metabolism (Supplementary Fig. 2).

**Figure 6:**
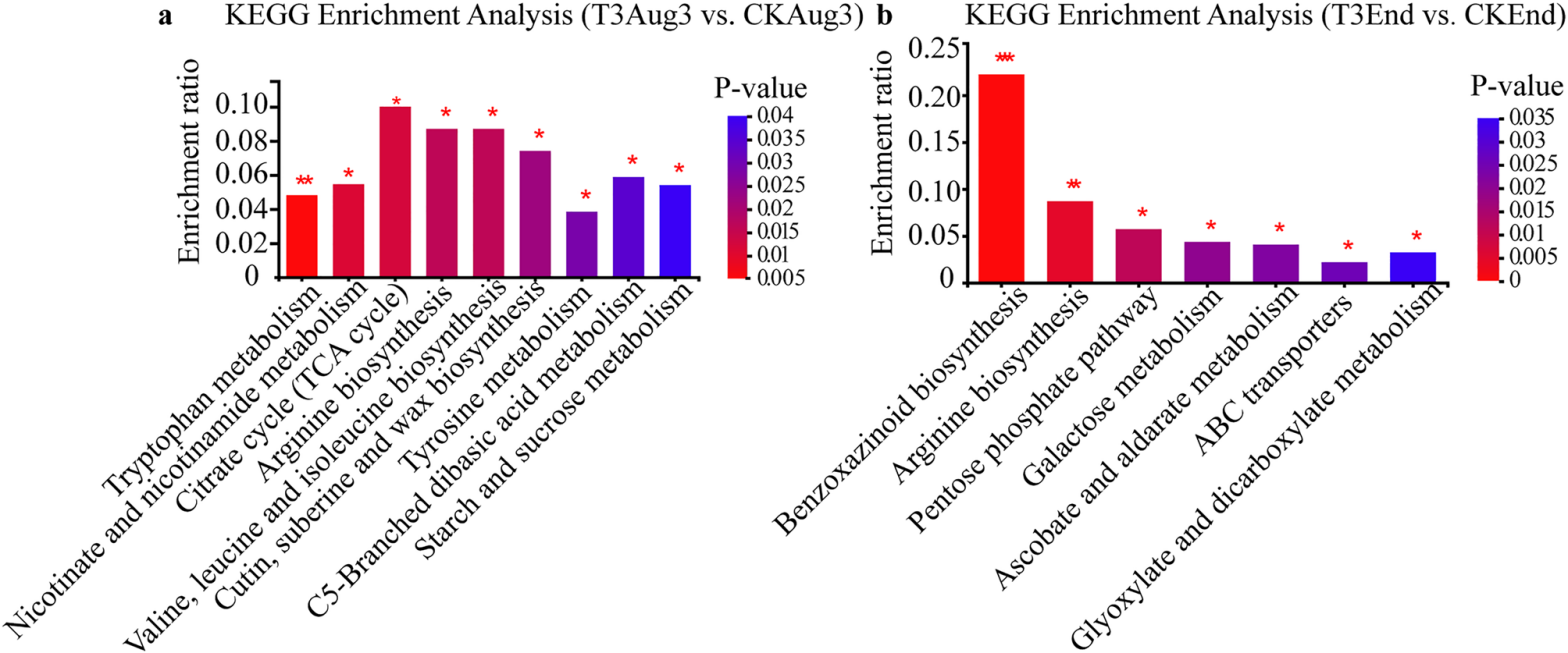
KEGG pathway enrichment analysis based on differentially abundant metabolites^21^. (**a**) T3Aug3 vs. CKAug3. (**b**) T3End vs. CKEnd. The horizontal coordinate is the name of the pathway, and the vertical coordinate indicates the enrichment ratio, which represents the ratio of the number of metabolites enriched in the pathway to the number of metabolites annotated to the pathway. T3Aug3 (samples from the T3 treatment on August 3rd), CKAug3 (samples from the CK treatment on August 3rd), T3End (samples from the T3 treatment on September 2nd) and CKEnd (samples from the CK treatment on September 2nd). A *P* value < 0.001 is marked ***, a *P* value < 0.01 is marked **, and a *P* value < 0.05 is marked *.

We annotated the important DAMs using the KEGG database and analyzed the KEGG pathways that were significantly enriched in DAMs (*P* < 0.05). In T3Aug3 vs. CKAug3, the significantly altered metabolic pathways included starch and sucrose metabolism, tryptophan metabolism and TCA (Fig. 6a). At the end of air-curing, the T3 treatment significantly altered benzoxazinoid biosynthesis, the pentose phosphate pathway and galactose metabolism (Fig. 6b).

#### Changes in DAMs in critical pathways

**Figure 7:**
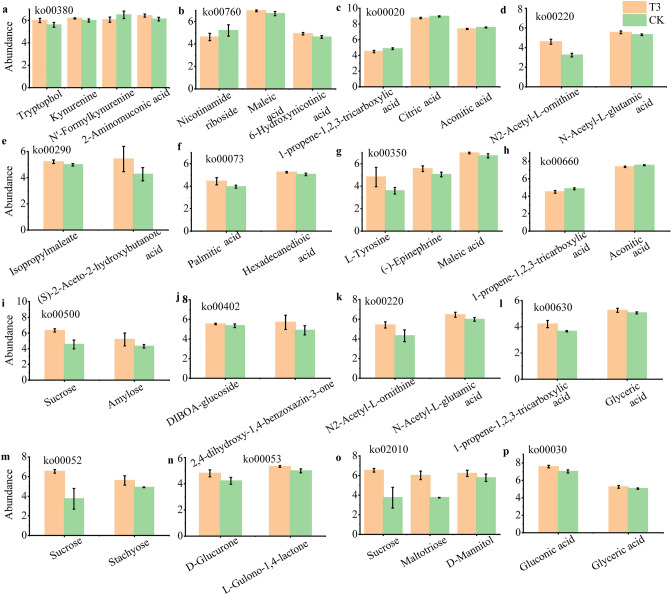
Analysis of differentially abundant metabolites in the significantly enriched KEGG pathway. (**a**–**i**) T3Aug3 vs. CKAug3. (**j**–**p**) T3End vs. CKEnd. T3Aug3 (samples from theT3 treatment on August 3rd), CKAug3 (samples from the CK treatment on August 3rd), T3End (samples from the T3 treatment on September 2nd) and CKEnd (samples from the CK treatment on September 2nd). All differences in the figures are significant (*P* < 0.05), and the vertical coordinates are mass spectral intensity values (mass spectral intensity after data preprocessing).

Moreover, the abundance of the DAMs in all significantly enriched KEGG pathways was analyzed in both comparison groups (Fig. 7), with pathway IDs corresponding to the pathway descriptions in Supplementary Table 3. In T3Aug3 vs. CKAug3, the abundance of the DAMs in the six significantly enriched pathways was higher in T3 than that in CK (Fig. 7a,b,d,e,f,g,i), and CK showed a higher relative abundance of DAMs (citric acid and 1-propene-1,2,3-tricarboxylic acid) in TCA cycle (Fig. 7c) and C5-branched dibasic acid metabolism (Fig. 7h) than that in T3.. In T3End vs CKEnd, the abundance of DAMs in all seven significantly enriched pathways showed T3 > CK (Fig. 7j–p). Among these KEGG enrichment pathways, N2-Acetyl-L-ornithine, L-tyrosine, N2-Acetyl-L-ornithine, sucrose and maltotriose were the five metabolites that were most significantly increased in abundance.

## Discussion

Normally, plant leaves gradually age due to the lack of sugars and sugar starvation, which further accelerates the breakdown of proteins and chlorophyll after harvesting^22,23^. Chlorophyll degradation massively occurs during plant senescence, and chloride a, chlorophyll alcohol, demagnetized chlorophyll, pheophytin a, NCCs (nonfluorescent chlorophyll catabolites), FCCs (fluorescent Chl catabolites) and RCCs (red Chl catabolites) are produced by chlorophyll degradation-related enzymes^24,25^. The green spots that occurred in the tobacco leaves of the wrapper variety CX-010 produced in the Enshi tobacco planting area directly impacted the consistency of the color of the tobacco leaves, thus affecting the availability of raw materials for cigars. In our study, spraying exogenous sucrose effectively limited the occurrence of green spots (Figs. 1,2). Although the fast appearance of green spots was accompanied by rapid water loss in all treatments, the water content of tobacco leaves in order was T3 < CK < T2 < T1, and the number of green spots in order was T3 < T2 < T1 < CK. It has been reported that sucrose possesses a moisturizing function at the appropriate concentrations^26,27^, which could be a possible explanation for why the T1 and T2 treatments had a higher total water content relative to the CK, which supported more chlorophyll degradation-related enzymes. However, the T3 treatment caused the largest limitation of green spot occurrence, which cannot be attributed to the change in the total water content. These results suggest that sucrose confers an unknown mechanism modulating the formation of green spots and that the total water content changes might be a result in sucrose concentration changes independent of green spot formation.

Zhang et al. reported that the TCA and polyphenols biosynthesis pathways were associated with the response of tobacco plants to various salinity conditions, and the DAMs mainly included tyrosine, tryptophan and critic acid^28^. In this study, we found that the DAMs classification certainly agreed with Zhang’s study, suggesting that these DAMs predominantly occupy the metabolic pathways in leaf senescence. Yu et al. identified three significantly differentially enriched KEGG pathways in the green spotted tissues compared to the normal tissues, including tyrosine metabolism, phenylalanine metabolism, and flavonoid biosynthesis, and the chlorogenic acid accumulated in green spots as a chemical marker of green spot formation^4^. Chlorogenic acid is the main phenolic substance in tobacco and forms brown pigments such as quinones through enzymatic browning reactions during the air-curing process to transform the color of leaves^29^. In the present study, we did not find a difference in chlorogenic acid between the T3 treatment and CK (Supplementary Tables 1,2). One possible reason is that the samples used for metabolomics analysis were whole leaf tissue, and green spotted tissue only accounted for a small part. Thus, the dilution effect should be carefully considered in explaining the results of the present study. Tyrosine is an aromatic amino acid that is a precursor to metabolites such as tryptophan and phenylalanine, which finally produce natural products, including pigments, alkaloids, and hormones^30,31^. It was reported that the addition of tyrosine modulated the development of beetroot leaves and increased the carotenoid content^32^. T3 treatment induced the accumulation of phenylpropanoids such as tyrosine, tryptophan and some flavonoids in tobacco leaves; thus, the sucrose-stimulated phenylpropanoids metabolism must play an important role in differentially regulating the senescence and formation of green spots on air-curing tobacco leaves. On the other hand, the TCA cycle is a core pathway involved in the metabolic process of fatty acid synthesis, glucose hypoplasia, transamination, and the synthesis of purines and pyrimidines^33,34^. Citric acid is the main intermediate metabolite of the TCA cycle, and Zahid et al. suggested that citric acid promoted plant growth and enhanced plant photosynthesis, thereby promoting chlorophyll formation^35^. All these results suggest that citric acid, tyrosine, and some metabolic pathways are involved in the conversion of pigments. In our study, T3 inhibited the accumulation of citric acid in the TCA cycle in tobacco leaves and promoted the accumulation of phenylpropanoids such as tyrosine and tryptophan. (Figs. 6,7). These results suggest that the reduced number of green spots in the exogenous sucrose treatments may be due to the changes in these substances and related metabolic pathways induced by sucrose spraying.

The air-curing tobacco leaves were deprived of external nutrient supply, and the energy required for their vital activities was only provided by respiration, which continuously decomposed and consumed their organic substances. A study of postharvest broccoli showed that sucrose treatment delayed decay, enhanced respiration, and provided an energy base for broccoli to stay fresh^36^. It has been suggested that the use of sugar-induced a rise in carbohydrates, the degradation of chlorophyll and the synthesis of anthocyanins in isolated leaves from *Egeria densa*^37^. These results suggest that the degradation of chlorophyll and the chloroplast requires a normal supply of energy and that sucrose can provide energy to the plant. Yu et al. concluded that insufficient protein degradation in green spots resulted in a significantly lower abundance of amino acids such as L-aspartic acid, L-phenylalanine, tyrosine, and DL-tryptophan in green spots than in normal air-curing tobacco^4^. In the present study, the addition of exogenous sucrose seemed to complement some of the energy required for the metabolism of the tobacco leaves, allowing the normal decomposition of proteins in the T3-treated tobacco leaves, which explains the generally higher amino acid and amino acid derivatives in the T3-treated tobacco leaves than in the CK-treated leaves (Fig. 7).

## Materials and methods

### Experimental materials

The trial was conducted in 2021 in Hejia village, Cuiba town, Enshi city, Hubei Province, China (E 109°47′42″, N 30°27′27"), at an altitude of 860 m. The cigar tobacco (*Nicotiana tabacum* L.) variety sampled was CX-010, a cultivated and promoted variety that originated from the germplasm bank of the Tobacco Research Institute of Hubei Province (Wuhan, China). Jinpeng Yang, Chunlei Yang, and Jun Yu of the Tobacco Research Institute of Hubei Province identified the CX-010 cigar tobacco, and with their permission, I collected plant samples. A voucher specimen of the plant used is deposited in the Huazhong Agricultural University. The planting site soil type was yellow brown soil. The basic physical and chemical properties of the soil at the trial site were as follows: pH 6.21, organic matter content 25.33 g/kg, available nitrogen content 126.05 mg/kg, available phosphorus content 83.34 mg/kg, available potassium content 535.19 mg/kg, and chloride ion content 0.11 mg/kg. The uniform young seedlings that germinated on seedling bowls were transplanted on May 11, with the row and plant spacing being 110 cm × 45 cm. After topping on July 10th, approximately 18 leaves remained. The application rates of fertilizers were 1,500 kg/hm^2^ of organic fertilizer and 120 kg/hm^2^ of pure N fertilizer, and P_2_O_5_ and K_2_O were calculated according to the ratio of N: P_2_O_5_: K_2_O = 1: 0.5: 3. All experiments were performed in accordance with local cigar production guidelines and regulations of the China National Tobacco Corporation Hubei Branch.

### Design of the experiment

On July 24th, more than 800 mature tobacco plants were available for harvesting, with the middle leaf (10th–14th leaf from the bottom) being harvested. The air-curing experiment was carried out in a closed steel air-curing room with controlled temperature and humidity. The air-curing room size was 12 m × 7 m × 7 m (length × width × height). The temperature and humidity of the air-curing room were controlled by humidifiers and exhaust fans. The humidity during the air-curing period was controlled as follows: 95 ± 3% during the withering period, 87 ± 3% during the color change period, 82 ± 3% during the color fixation period, and 70 ± 3% during the stem-drying stage period. The temperature of the air-curing room was controlled between 24 and 28°C during the air-curing period.

**Figure 8:**
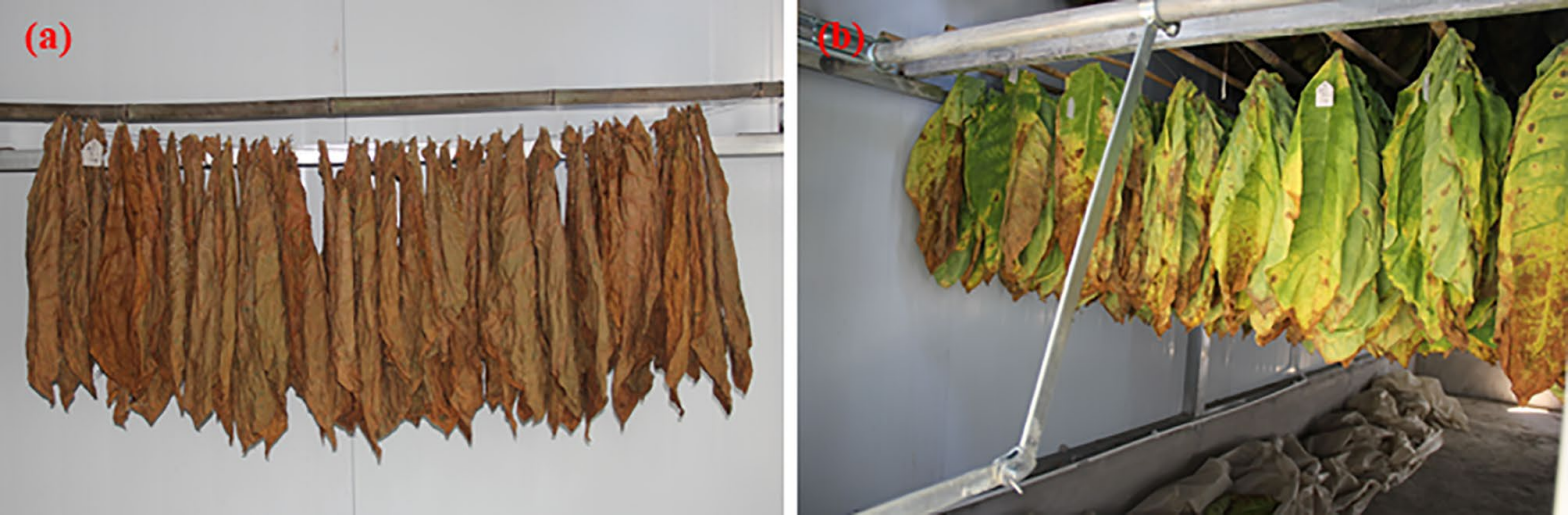
The distribution of tobacco leaves during air-curing.

The selected tobacco leaves were hung in the air-curing room with 4 treatments: T1 (0.1 M sucrose), T2 (0.2 M sucrose), T3 (0.4 M sucrose) and CK (pure water), with 3 replicates for each treatment. Approximately 50 leaves were hung on a bamboo stick with 160 cm in length (Fig. 8a), and 3 sticks composed a replicate. A 25 cm spacing between adjacent sticks was used (Fig. 8b), and the replicates for each treatment were randomly arranged. The sucrose solution was sprayed evenly on each tobacco air-curing leaf (front and back), and 2 L solution was sprayed on the leaves on 9 sticks (approximately 5 mL for each leaf).

Three leaves from each stick were marked with white tags to investigate green spot variation during air-curing, and a sample of 5 leaves was randomly sampled from each replicate for investigating the other traits at each time point. During the air-curing period, samples were taken every 5 days for a total of 9 times from July 24th to September 2nd. After sampling at each time point, the main veins of leaves were removed. Half of the samples were then used to measure the total water content, and the other half were taken back to the laboratory and placed in a − 80°C refrigerator for metabolomics analysis.

### Measurement items and methods

#### Determination of the total water content

When the air-curing tobacco was sampled, it was immediately weighed and recorded as fresh weight (FW). Then the sample was dried at 65 °C to a steady weight in a blast drying box, and the weights were recorded as dry weight (DW). The experiment was repeated three times.

$${\text{Total}}\,{\text{water}}\,{\text{content}}\,{\text{of}}\,{\text{tobacco}}\,{\text{leaves}}\left( \% \right) = [({\text{FW}} - {\text{DW}}) \div {\text{FW}}] \times 100\%$$

#### Measurement of green spots

The number and size of green spots on tobacco leaves were counted at the same time as sampling during the air-curing period. Three leaves from three sticks were marked at the beginning of air curing, including the 5th leaf near the middle on both sides and one in the middle, for a total of 9 leaves per treatment. Each time, these 9 leaves were used for the green spot survey. Three sticks of tobacco were fixed for each treatment, with three leaves per stick. The average of the number of green spots of the three leaves represented the green spot occurrence of one stick, and the average of the number of green spots of the three sticks represented the green spot occurrence of one treatment. The size of green spots of tobacco leaves was measured with Vernier calipers, and the spots were classified into diameter (d) ≤ 2 mm, 2 mm < d ≤ 5 mm, 5 mm < d ≤ 10 mm, and d > 10 mm according to their maximum diameter.

#### Microstructure observation

The air-curing tobacco leaves were fixed with fixative (70% ethanol) for more than 24 h. The samples were dehydrated with a concentration gradient of alcohol and then embedded in paraffin and sliced (4 μm thick) for observation of the tissue structure of tobacco leaves.

The air-curing tobacco leaves were cut into 1 mm × 2 mm segments and quickly placed in a weighing bottle containing 2.5% glutaraldehyde fixative (pH 7.2). Suction was performed with a syringe, so that the material was fixed as soon as possible in a 4°C refrigerator. Then, 1% osmic acid prepared with 0.1 M phosphate buffer (pH 7.4) was fixed at room temperature for 7 h in the dark, and 0.1 M phosphate buffer (pH 7.4) was used to rinse the samples three times for 15 min each time. The fixed samples were dehydrated with gradient ethanol propanol (acetone washing dehydration), embedded in epoxy resin Epon-812, and sliced by an ultrathin sectioning machine (Leica UC7, Germany). The sections were stained with uranium acetate-lead citrate solution, fixed to copper mesh after citric acid staining, and finally observed and photographed by transmission electron microscopy (Hitachi H-7650, Japan) at a working voltage of 100 kV.

### Metabolomics processing

#### Metabolite extraction

The following experimental procedure is based on the operation procedure of Majorbio^38^. Fifty milligrams of sample was accurately weighed, and the metabolites were extracted using a 400 µL methanol:water (4:1, v/v) solution with 0.02 mg/mL L-2-chlorophenylalanin as an internal standard. The mixture was allowed to settle at − 10°C and treated with a high-throughput tissue crusher Wonbio-96c (Shanghai Wanbai Biotechnology Co., Ltd.) at 50 Hz for 6 min, then followed by ultrasound at 40 kHz for 30 min at 5°C. The samples were placed at – 20°C for 30 min to precipitate proteins. After centrifugation at 13,000*g* at 4°C for 15 min, the supernatant was carefully transferred to sample vials for LC‒MS/MS analysis.

#### Quality control samples 

As a part of the system conditioning and quality control process, a pooled quality control sample (QC) was prepared by mixing equal volumes of all samples. The QC samples were disposed and tested in the same manner as the analytic samples and measured every 8th sample.

#### LC‒MS/MS analysis

The instrument platform for LC**‒**MS analysis was the Q-Exactive Orbitrap mass analyzer of Thermo Fisher Scientific.

Chromatographic conditions: 2 μL of sample was separated by an HSS T3 column (100 mm × 2.1 mm i.d., 1.8 μm) and then subjected to mass spectrometry detection. The mobile phases consisted of 0.1% formic acid in water: acetonitrile (95:5, v/v) (solvent A) and 0.1% formic acid in acetonitrile: isopropanol: water (47.5:47.5:5, v/v) (solvent B). The chromatographic gradient elution procedure is shown in Supplementary Table 4. The sample injection volume was 2 µL and the flow rate was set to 0.4 mL/min. The column temperature was maintained at 40°C. During the period of analysis, all these samples were stored at 4°C.

MS conditions: The mass spectrometric data were collected using a Thermo UHPLC-Q Exactive Mass Spectrometer equipped with an electrospray ionization (ESI) source operating in either positive or negative ion mode. The optimal conditions were set as follows: heater temperature, 400°C; capillary temperature, 320°C; sheath gas flow rate, 40 arb; aux gas flow rate, 10 arb; ion-spray voltage floating (ISVF), − 2800 V in negative mode and 3500 V in positive mode; and normalized collision energy, 20–40–60 V rolling for MS/MS. Full MS resolution was 70,000, and MS/MS resolution was 17,500. Data acquisition was performed in data-dependent acquisition (DDA) mode. Detection was carried out over a mass range of 70–1050 m/z.

#### Data preprocessing and annotation

After mass spectrometry detection was completed, the raw data of LC/MS was preprocessed by Progenesis QI (Waters Corporation, Milford, USA) software, and a three-dimensional data matrix in CSV format was exported. The information in this three-dimensional matrix includes sample information, metabolite name and mass spectral response intensity. Internal standard peaks, as well as any known false-positive peaks (including noise, column bleed, and derivatized reagent peaks), were removed from the data matrix, with redundant data removed and peaks pooled. At the same time, the metabolites were searched and identified, and the main databases were the HMDB (http://www.hmdb.ca/), Metlin (https://metlin.scripps.edu/) and Majorbio Database.

The data from the database search were uploaded to the Majorbio cloud platform (https://cloud.majorbio.com) for data analysis. Metabolic features detected in at least 80% across all sets of samples were retained. After filtering, minimum metabolite values were imputed for specific samples in which the metabolite levels fell below the lower limit of quantitation, and each metabolic features was normalized by summation. To reduce the errors caused by sample preparation and instrument instability, the response intensities of the sample mass spectrometry peaks were normalized by summation to obtain a normalized data matrix. Moreover, variables with a relative standard deviation (RSD) > 30% in the QC samples were removed, and log10 normalized to obtain the final data matrix for subsequent analysis.

#### Analysis of DAMs

The variance analysis was performed on the matrix file after data preprocessing. The R package ropls (Version 1.6.2) was used to perform principal component analysis (PCA) and orthogonal least partial squares discriminant analysis (OPLS-DA), and 7-cycle interactive validation was used to evaluate the stability of the model. In addition, Student’s t test and fold difference analysis were performed. The selection of significantly different metabolites was determined based on the variable importance in the projection (VIP) obtained by the OPLS-DA model and the *P* value of Student’s t test, and the metabolites with a VIP > 1 and *P* < 0.05 were considered significant DAMs. DAMs among the two groups were summarized and mapped into their biochemical pathways through metabolic enrichment and pathway analysis based on a database search (KEGG, http://www.genome.jp/kegg/). These DAMs were classified according to the pathways they were involved in or the functions they performed. Scipy.stats (Python packages) (https://docs.scipy.org/doc/scipy/) was exploited to identify statistically significantly enriched pathways using Fisher’s exact test.

### Measurement items and methods

All experimental results are expressed as the mean values ± standard deviations (mean ± sd). Data were collated using Microsoft Excel 2016. Charts were produced by Origin 2021, and Adobe Illustrator (2020) was used for the layout of images.

## Conclusion

In the present study, exogenous sucrose spraying alleviated the occurrence of green spots in tobacco leaves during the air-curing period. Metabolomic analysis revealed that exogenous sucrose altered the abundance of key metabolites, such as tyrosine and citric acid, as well as metabolic pathways, including amino acid metabolism and the TCA cycle. Therefore, these metabolites are likely to be involved in the appearance of green spots and exogenous sucrose can be applied to manage the occurrence of green spots in tobacco leaves during air-curing.

## Supplementary Information

Supplementary Information 1.

Supplementary Information 2.

Supplementary Information 3.

Supplementary Information 4.

Supplementary Information 5.

Supplementary Information 6.

## Data Availability

The datasets generated during the current study are available from the corresponding author on reasonable request.
