# Supplementary material for: Metabolomic analysis reveals key metabolites alleviating green spots under exogenous sucrose spraying in air-curing cigar tobacco leaves
Source: Sci Rep. 2023 Jan 24;13:1311. doi: 10.1038/s41598-023-27968-8 (PMC9873923; doi:10.1038/s41598-023-27968-8)
Supplement: Supplementary file 1 — Supplementary Information 1. [file 41598_2023_27968_MOESM1_ESM.docx]

Metabolomic analysis reveals key metabolites alleviating green spots under exogenous sucrose spraying in air-curing cigar tobacco leaves

Nanfen Li^1^, Jun Yu^2^, Jinpeng Yang^2*^, Sheliang Wang^1^, Lianying Yu^1^, Fangsen Xu^1*^, Chunlei Yang^2*^

^1^Microelement Research Center, College of Resource and Environment, Huazhong Agricultural University, Wuhan, China

^2^Tobacco Research Institute of Hubei Province, Wuhan, China

***Correspondence:** Fangsen Xu (fangsenxu@mail.hzau.edu.cn); Jinpeng Yang (yjp2022@162.com); Chunlei Yang (ycl193737@163.com)


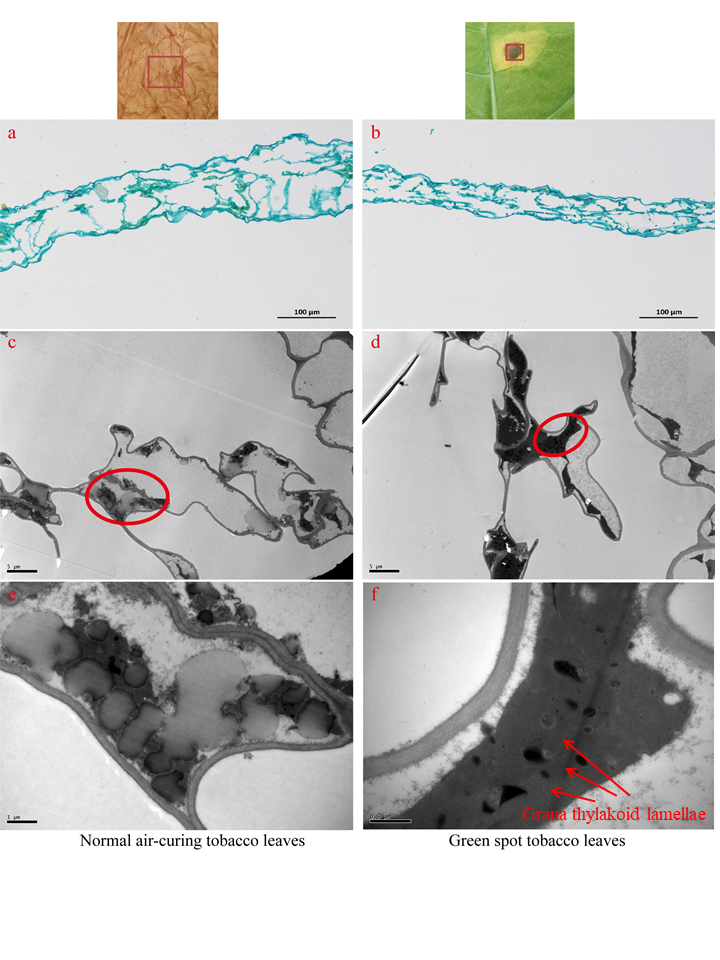


**Supplementary Figure 1.** Comparison of microstructure of green spot and normal tobacco leaves. The left column is the normal air-curing tobacco leaves, including (**a**) the tissue structure, (**c**) the ultrastructure and (**e**) the enlargement of the red circle in (**c**). The right column is the tissue of green spot tobacco leaves, including (**b**) the tissue structure, (**d**) the ultrastructure and (**f**) the enlargement of the red circle in of (**d**).
