# Supplementary material for: Metabolomic analysis reveals key metabolites alleviating green spots under exogenous sucrose spraying in air-curing cigar tobacco leaves
Source: Sci Rep. 2023 Jan 24;13:1311. doi: 10.1038/s41598-023-27968-8 (PMC9873923; doi:10.1038/s41598-023-27968-8)
Supplement: Supplementary file 2 — Supplementary Information 2. [file 41598_2023_27968_MOESM2_ESM.docx]

Metabolomic analysis reveals key metabolites alleviating green spots under exogenous sucrose spraying in air-curing cigar tobacco leaves

Nanfen Li^1^, Jun Yu^2^, Jinpeng Yang^2*^, Sheliang Wang^1^, Lianying Yu^1^, Fangsen Xu^1*^, Chunlei Yang^2*^

^1^Microelement Research Center, College of Resource and Environment, Huazhong Agricultural University, Wuhan, China

^2^Tobacco Research Institute of Hubei Province, Wuhan, China

***Correspondence:** Fangsen Xu (fangsenxu@mail.hzau.edu.cn); Jinpeng Yang (yjp2022@162.com); Chunlei Yang (ycl193737@163.com)


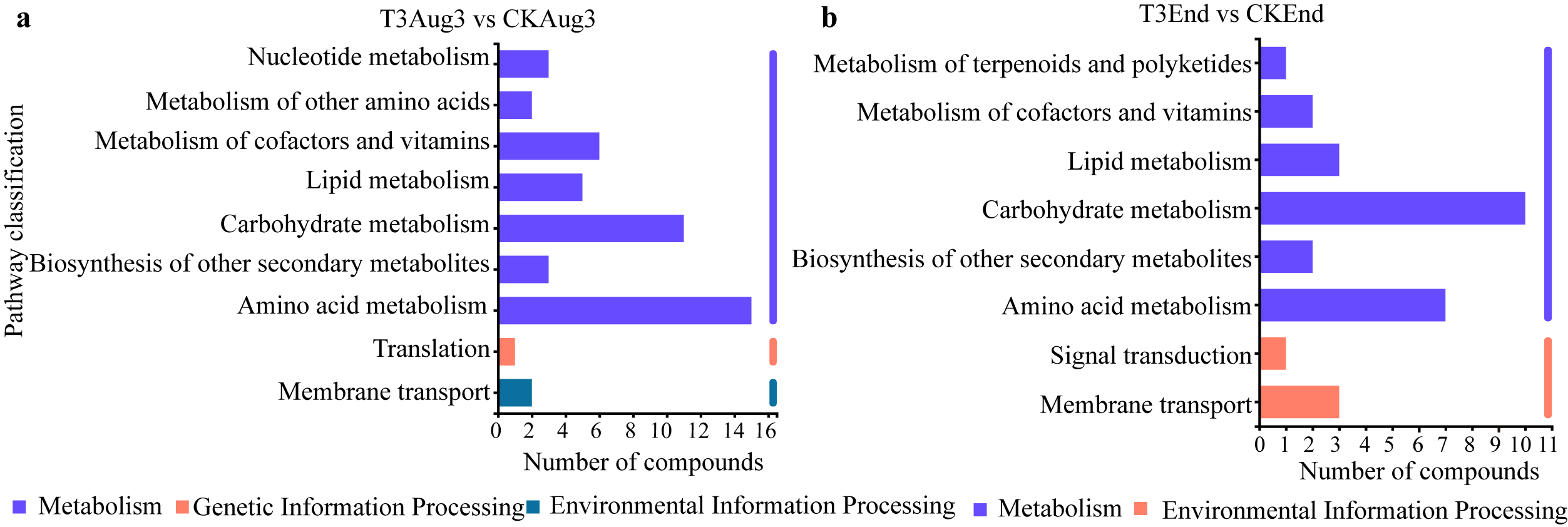


**Supplementary Figure 2.** KEGG pathway function statistics. (**a**) and (**b**) are T3Aug3 vs. CKAug3 and T3End vs. CKEnd, respectively. T3Aug3 (samples from the T3 treatment on August 3rd), CKAug3 (samples from the CK treatment on August 3rd), T3End (samples from the T3 treatment on September 2nd) and CKEnd (samples from the CK treatment on September 2nd).
